# Low Pressure Radio-Frequency Oxygen Plasma Induced Oxidation of Titanium – Surface Characteristics and Biological Effects

**DOI:** 10.1371/journal.pone.0084898

**Published:** 2013-12-26

**Authors:** Wan-Yu Tseng, Sheng-Hao Hsu, Chieh-Hsiun Huang, Yu-Chieh Tu, Shao-Chin Tseng, Hsuen-Li Chen, Min-Huey Chen, Wei-Fang Su, Li-Deh Lin

**Affiliations:** 1 School of Dentistry, National Taiwan University, Taipei, Taiwan; 2 Department of Dentistry, National Taiwan University Hospital, Taipei, Taiwan; 3 Institute of Polymer Science and Engineering, National Taiwan University, Taipei, Taiwan; 4 Department of Materials Science and Engineering, National Taiwan University, Taipei, Taiwan; Massey University, New Zealand

## Abstract

**Objective:**

This research was designed to investigate the effects of low pressure radio-frequency (RF) oxygen plasma treatment (OPT) on the surface of commercially pure titanium (CP-Ti) and Ti6Al4V. Surface topography, elemental composition, water contact angle, cell viability, and cell morphology were surveyed to evaluate the biocompatibility of titanium samples with different lengths of OP treating time.

**Materials and Methods:**

CP-Ti and Ti6Al4V discs were both classified into 4 groups: untreated, treated with OP generated by using oxygen (99.98%) for 5, 10, and 30 min, respectively. After OPT on CP-Ti and Ti6Al4V samples, scanning probe microscopy, X-ray photoelectron spectrometry (XPS), and contact angle tests were conducted to determine the surface topography, elemental composition and hydrophilicity, respectively. The change of surface morphology was further studied using sputtered titanium on silicon wafers. 3-[4,5-dimethylthiazol-2-yl]-2,5-diphenyltetrazolium bromide (MTT) assay and F-actin immunofluorescence stain were performed to investigate the viability and spreading behavior of cultivated MG-63 cells on the samples.

**Results:**

The surface roughness was most prominent after 5 min OPT in both CP-Ti and Ti6Al4V, and the surface morphology of sputtered Ti sharpened after the 5 min treatment. From the XPS results, the intensity of Ti^°^, Ti^2+^, and Ti^3+^ of the samples’ surface decreased indicating the oxidation of titanium after OPT. The water contact angles of both CP-Ti and Ti6Al4V were increased after 5 min OPT. The results of MTT assay demonstrated MG-63 cells proliferated best on the 5 min OP treated titanium sample. The F-actin immunofluorescence stain revealed the cultivated cell number of 5 min treated CP-Ti/Ti6Al4V was greater than other groups and most of the cultivated cells were spindle-shaped.

**Conclusions:**

Low pressure RF oxygen plasma modified both the composition and the morphology of titanium samples’ surface. The CP-Ti/Ti6Al4V treated with 5 min OPT displayed the roughest surface, sharpest surface profile and best biocompatibility.

## Introduction

Osseointegration is the direct structural and functional connection between living bone tissue and the surface of dental implants. This is recognized as a direct bone-implant contact without any intervening connective tissue layers [1,2]. Besides the biocompatibility of the implant materials per se, the surface composition, structure, topography, oxide thickness, roughness and surface contaminants also influence the cells’ behaviors [3-6]. Titanium was found to have good biocompatibility. Its osseointegration layer is a naturally-formed, thin and adherent surface oxide layer [7,8]. To further improve the osseointegration on titanium based implants, the methods to form the titanium oxide of designed thickness, chemical composition and topography were researched, tested and developed [9-14].

Plasma, the fourth state of matter, consists of neutral atoms, electrons, ions and radicals, on average is electrical neutral [15,16]. Since the energetic ionized particles and radicals are highly reactive, different plasma technologies have been widely used for the surface pretreatment or cleaning of semiconductors and metals [17-20]. Therefore, plasma technology can be also used to clean titanium surfaces thereby improving its biocompatibility [6,21]. Besides, the gas plasma generated by low pressure radio-frequency (RF) devices performs good conformity between the electrodes, which overcomes the surface structure of high aspect ratio to achieve isotropic surface treatment. Hence, low pressure RF gas plasma is widely applied on the processing of fibers, microfluid devices, biomedical devices and etc [22-26]. We have already utilized low pressure RF oxygen plasma (OP) to expose silica nanoparticles on the surface of polyacrylate/silica nanocomposite. We then fabricated a superhydrophobic and oleophobic transparent nanocomposite by grafting fluorosilane onto the exposed silica nanoparticles via the sol-gel process. In our previous study, oxygen plasma treatment (OPT) has shown effective oxidation ability with long range (in centimeter scale) uniformity [27]. Thus, OPT is an effective surface modification technique, which can modify physicochemical properties, including hydrophilicity/hydrophobicity, and improve the surface properties of materials. Therefore, appropriate plasma processes can also improve the adhesion of biocompatible materials to tissue [21].

In this study, we sought to evaluate the surface properties and biocompatibility of commercially pure titanium (CP-Ti) and the alloy, Ti6Al4V before and after OPT for different lengths of time. Low pressure RF OP generated at room temperature was used in this study. The hypothesis was that surface properties, such as contact angle and surface topography, would be changed after surface treatment by OP for different lengths of time and in addition, the biocompatibility would improve.

## Materials and Methods

### Sample Preparation

Metal disk samples, 16 mm in diameter and 2 mm in thickness, were cut from grade 4 CP-Ti and grade 5 Ti6Al4V sticks (both from SPEMET CO. Ltd., Taipei, Taiwan). The surface of each sample was polished by flat lapping (Young-Wing Co. Ltd., New Taipei City, Taiwan) to obtain a mirror-like surface. Each disc sample was cleaned by n-hexane (97%, Acros, Geel, Belgium), 95% ethanol (Acros, Geel, Belgium) and distilled water sequentially. After sample cleaning, the water on the sample surface was wiped off and the samples were vacuum dried (1×10^-3^ torr) for 4 hours. A thin film of 100 nm Ti (sputtered Ti) was deposited on a Si wafer using a 3 inch titanium target (Summit-tech, Hsinchu, Taiwan) and a home-made sputter (Yuan-Shian Vacuum, New Taipei City, Taiwan) operated at room temperature at 1×10^-3^ torr with the deposition rate fixed at 0.3 nm‧s^-1^. To carry out oxygen plasma treatment (OPT), the samples were put into the quartz chamber of the plasma cleaner (Harrick Plasma, NY, USA) and treated with OP. The oxygen pressure of 5~6×10^-1^ torr and power of 18 watts were adopted to generate OP in the quartz chamber. Three kinds of samples, CP-Ti, Ti6Al4V and sputtered Ti, were classified into 4 groups of samples including: untreated (control) and 5, 10 and 30 min OPT groups (OPT(5), OPT(10), and OPT(30)), respectively. 

### Surface Characterization

An atomic force microscope (MultiMode SPM, Veeco, Santa Barbara, USA) was operated in tapping mode to probe the surface roughness and morphological changes of disc samples before and after difference lengths of OP treatment time. The root mean square (RMS) roughness of five areas (1 μm × 1 μm) on each sample were observed and averaged. One sample for each substrate was adopted for measurement. Every five scans (512 scan lines for each scan) were conducted to determine roughness of the same sample before and after OPT for 5, 10, and 30 min, respectively. The spectra of X-ray photoelectron spectrometry (XPS) (ULVAC-PHI, Chigasaki, Japan) were obtained by using Al *K*_*α*_ X-ray source with a photoelectron take-off angle of 45° at a high vacuum environment (1×10^-7^ torr) to examine core-levels. The adopted beam size was 100 μm. The hydrophilicity of CP-Ti and Ti6Al4V samples before and after plasma treatment was determined using a contact angle analyzer (Sindatek Model 100SB, Sindatek, Taipei, Taiwan). Distilled water was used in the measurement.

### Measurement of Cytotoxicity

MG-63 (CRL-1427, ATCC), a human osteosarcoma cell line, was used for this experiment [28]. The cells were cultured in Dulbecco's modified Eagle’s medium (DMEM, Gibco BRL, Gaithersburg, MD) with 10% fetal bovine serum (FBS, Gibco BRL), 100 U/mL penicillin (Gibco BRL), and 100 mg/mL streptomycin (Gibco BRL) at 37 °C in the incubator in 5% CO_2_ and 100% humidity.

The OP treated or untreated CP-Ti or Ti6Al4V metal discs were placed in a 24-well plate with 10^4^ cells per well. The cellular activities of MG-63 on various samples were compared after 7 days by an assay based on the reduction of the tetrazolium salt 3-[4,5-dimethylthiazol-2-yl]-2,5- diphenyltetrazoliumbromide (MTT) [29-31]. Briefly, the cells were incubated with fresh medium containing 0.5 mg/ml MTT for 2 hours. The cells with functional mitochondria can reduce MTT to an insoluble purple formazan product. The medium was then aspirated and the reduced formazan was dissolved with DMSO. Their optical density (OD 570) was measured with a Dias Microwell Plate Reader (Dynatech Medical Products, Ltd, St. Peter Port, UK) and the absorbance was taken as proportional to the number of viable cells present (n=5).

### Immunofluorescence stain of the F-actin cytoskeleton

The F-actin cytoskeleton in MG-63 was observed by using an immunofluorescence stain at the 7^th^ day of cultivated cells on CP-Ti and Ti6Al4V metal discs. After an appropriate period of experimentation, the MG-63 cells were fixed with 4% paraformaldehyde in PBS for 20 min, and washed with PBS containing 0.05% Tween-20 twice. Subsequently, the cells was permeabilized with 0.1% Triton X-100 in PBS for 5 min, washed with PBS twice and blocked with 1% BSA for 30 min. TRITC conjugated Phalloidin at a concentration of 1:1000 was used as the primary antibody and incubated with cells for 1 hr. Then, 4',6-diamidino-2- phenylindole (DAPI) was used to counterstain cellular nuclei. Finally, the stained cells were visualized with a fluorescence microscope.

### Statistical analysis

Surface roughness and water contact angle were analyzed using ANOVA and then tested with post-hoc Tukey/Kramer test with *p* < 0.05 being considered significant. Results from MTT assays are expressed as a percentage of the control groups. Statistically significant differences for each variable were established using ANOVA and the post-hoc Tukey/Kramer test with *p* < 0.05 being considered significant.

## Results

**Figure 1:**
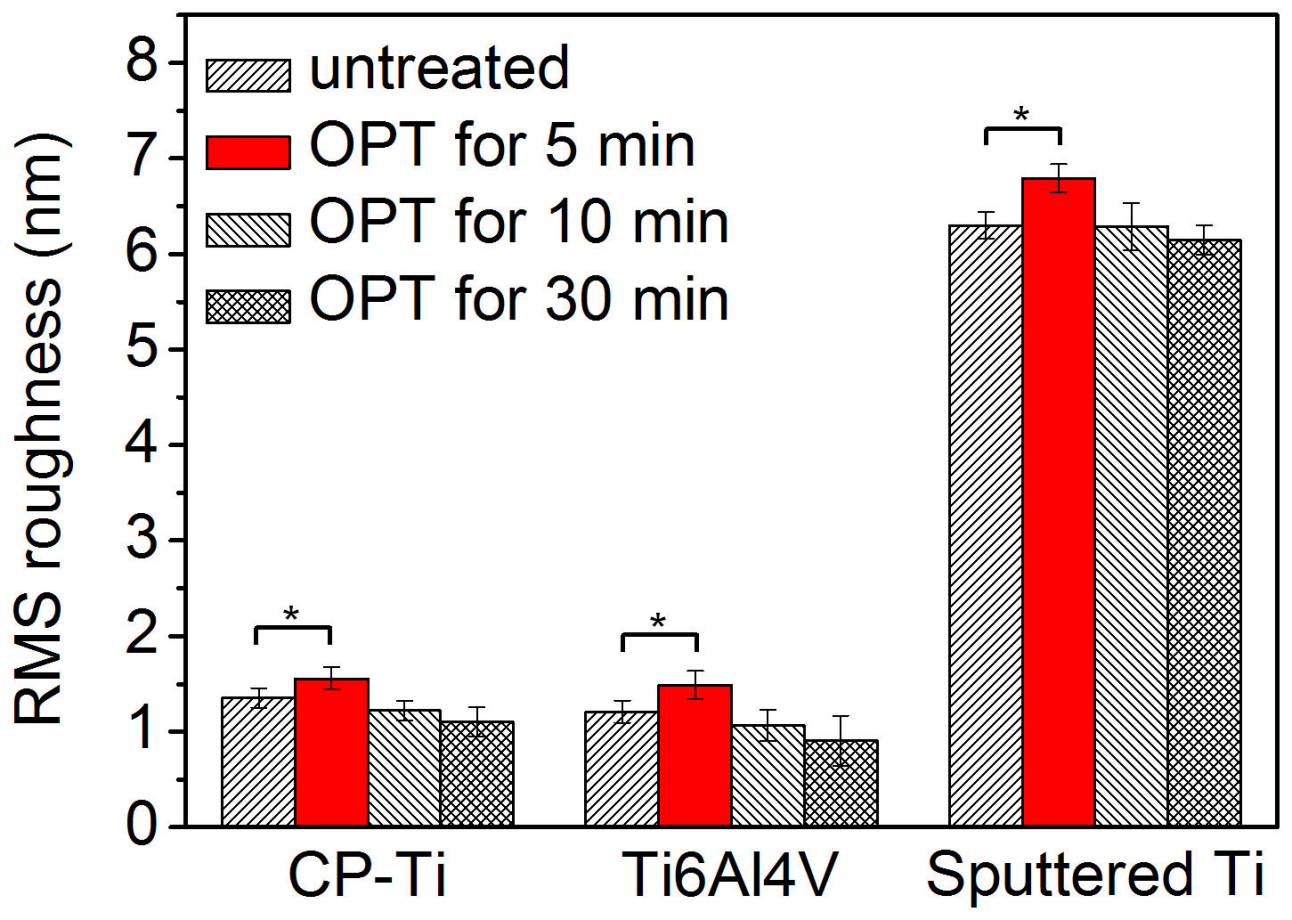
Surface roughness of CP-Ti, Ti6Al4V and sputtered Ti specimen after oxygen plasma treatment for different lengths of time, respectively. Star sign means significant difference (*p* < 0.05).

**Figure 2:**
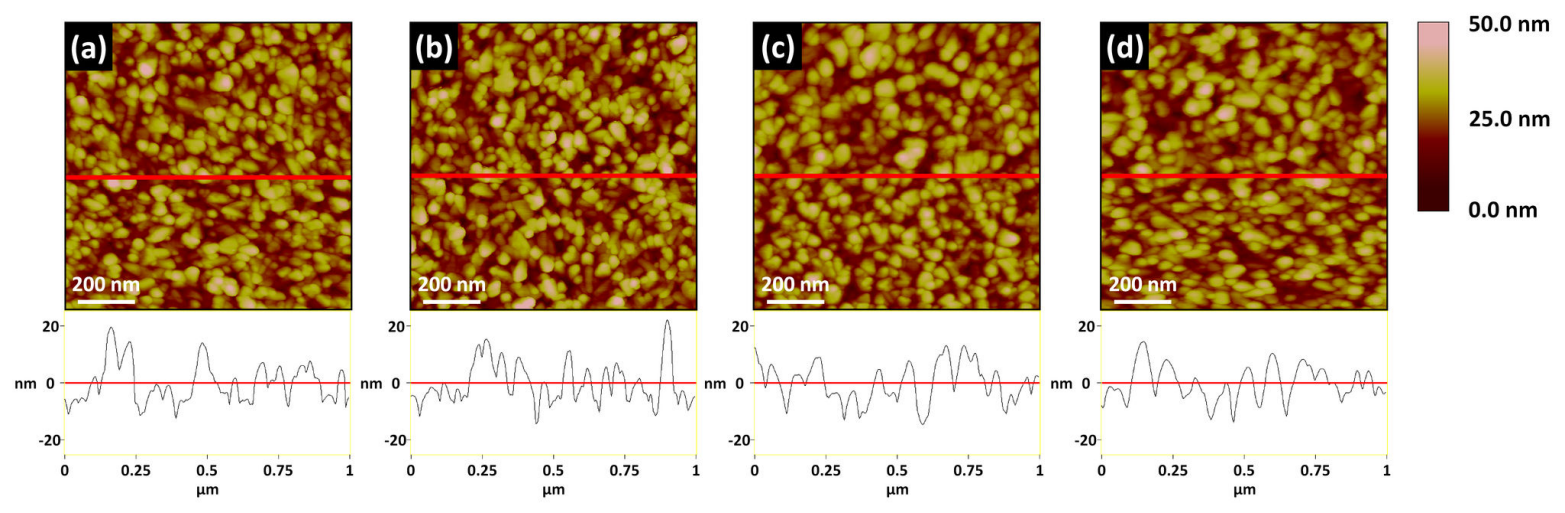
Topographic images with section analysis of sputtered Ti substrates: (a) untreated, (b) OPT for 5 minutes, (c) OPT for 10 minutes and (d) OPT for 30 minutes.

RMS roughness of various surfaces is shown as the mean with the standard deviation in Figure 1. The RMS roughness of the CP-Ti sample in the control, OPT(5), OPT(10), and OPT(30) groups are 1.35 (±0.10), 1.56 (±0.12), 1.22 (±0.10) and 1.10 (±0.15) nm, respectively. For the Ti6Al4V group, the roughness was 1.21 (±0.12), 1.49 (±0.15), 1.07 (±0.16) and 0.91 (±0.27) nm in the control, OPT(5), OPT(10), and OPT(30) groups, respectively. The RMS roughness of sputtered Ti on the control, OPT(5), OPT(10), and OPT(30) groups was 6.29 (±0.14), 6.79 (±0.15), 6.29 (±0.25) and 6.14 (±015) nm, respectively. All three substrate groups increased their roughness after 5 min OPT and then the roughness decreased after OPT for a longer time. Figure 2 shows the topographic images and section analysis of the sputtered Ti substrate. The domain size of the granules on sputtered Ti surface seems to have increased when the treatment time increased. From the section analysis, the surface morphology of sputtered Ti was sharpened in OPT(5); however, the sharpened structure was then smoothed down after a longer time of treatment.

**Figure 3:**
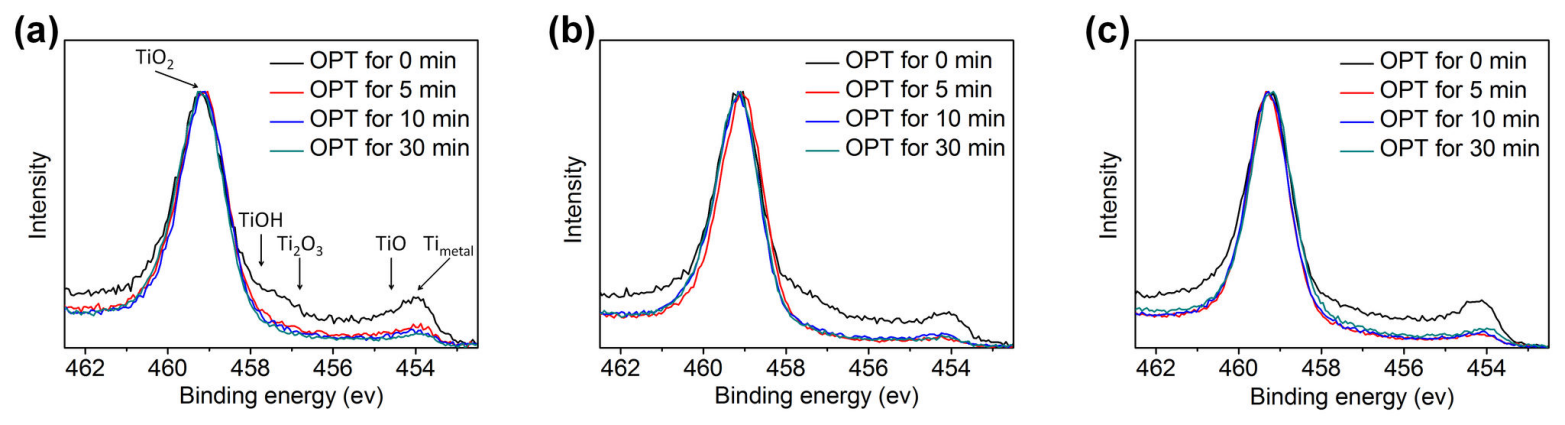
Normalized XPS spectra of (a) CP-Ti, (b) Ti6Al4V and (c) sputtered Ti before and after oxygen plasma treatment for different lengths of time.

The normalized Ti XPS spectra of CP-Ti, Ti6Al4V and sputtered Ti treated by OP for different lengths of time are shown in Figure 3 (a), (b) and (c), respectively. The Ti 2p_3/2_ peak intensity is attributed to Ti_metal_ (454 ev), TiOH (457.5 ev), TiO (454.6 ev), Ti_2_O_3_ (456.8 ev) and TiO_2_ (459.0 ev). As compared with the untreated sample, the intensity of Ti_metal_, TiOH, TiO and Ti_2_O_3_ decreased which demonstrates that OPT contributes to TiO_2_ forming on the surface of titanium samples by oxidation [32-34]. However, the Ti 2p_3/2_ spectra had almost no difference when treatment time was longer than 5 min for each material. 

**Figure 4:**
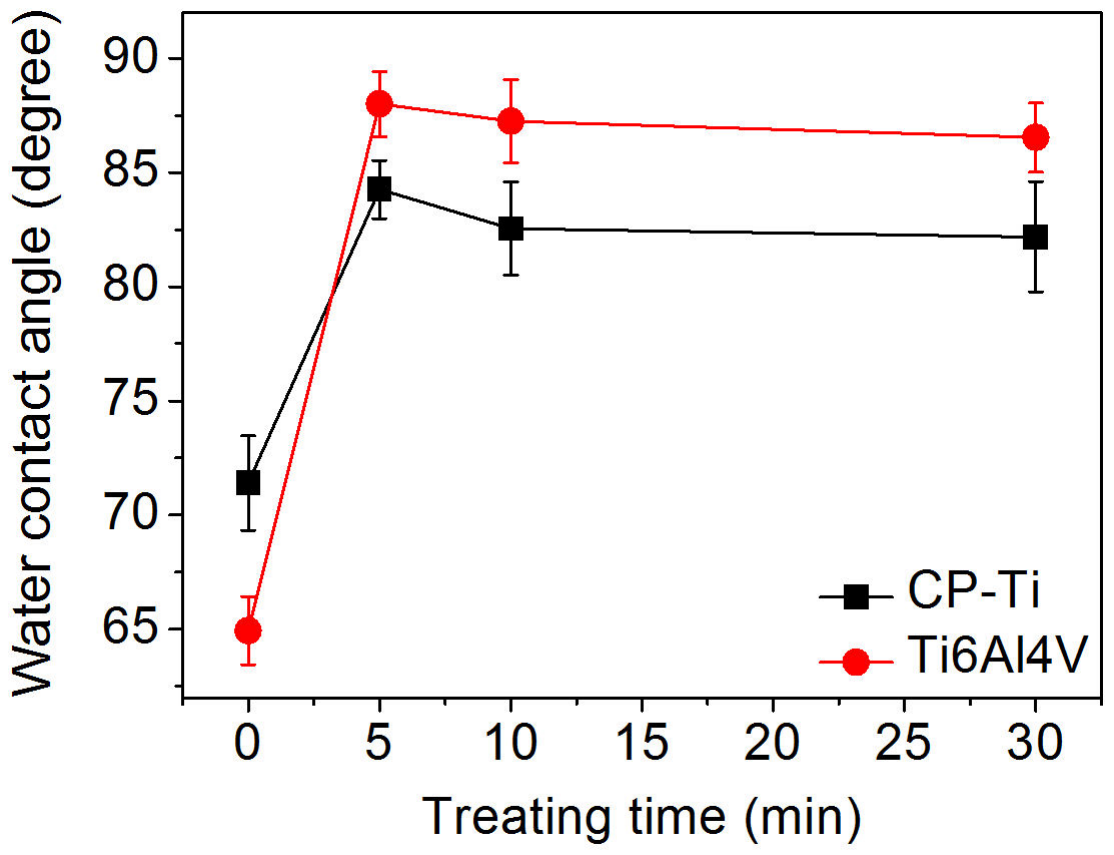
Water contact angles of CP-Ti and Ti6Al4V specimen treated by oxygen plasma.

The results of the water contact angle test on the CP-Ti and Ti6Al4V samples shown in Figure 4 display similar phenomenon to the change of surface composition. The water contact angle of untreated CP-Ti and Ti6Al4V were 71.4 (±2.07)° and 64.94 (±1.49)°, respectively. After OPT for 5 min, the water contact angle of both CP-Ti and Ti6Al4V increased. The water contact angles of CP-Ti in the OPT(5), OPT(10), and OPT(30) groups were 84.26 (±1.27), 82.54 (±2.04) and 82.18 (±2.42)°, respectively. For Ti6Al4V, the water contact angles were 88.00 (±1.42), 87.24 (±1.82) and 86.54 (±1.51)° in OPT(5), OPT(10), and OPT(30) groups, respectively. There was no significant difference in the contact angle among the samples treated by OP for longer than 5 min. Overall, there was no difference among the samples treated by OP for longer than 5 min on the hydrophilicity and the Ti 2p_3/2_ intensity of different titanium valence states determined by XPS.

**Figure 5:**
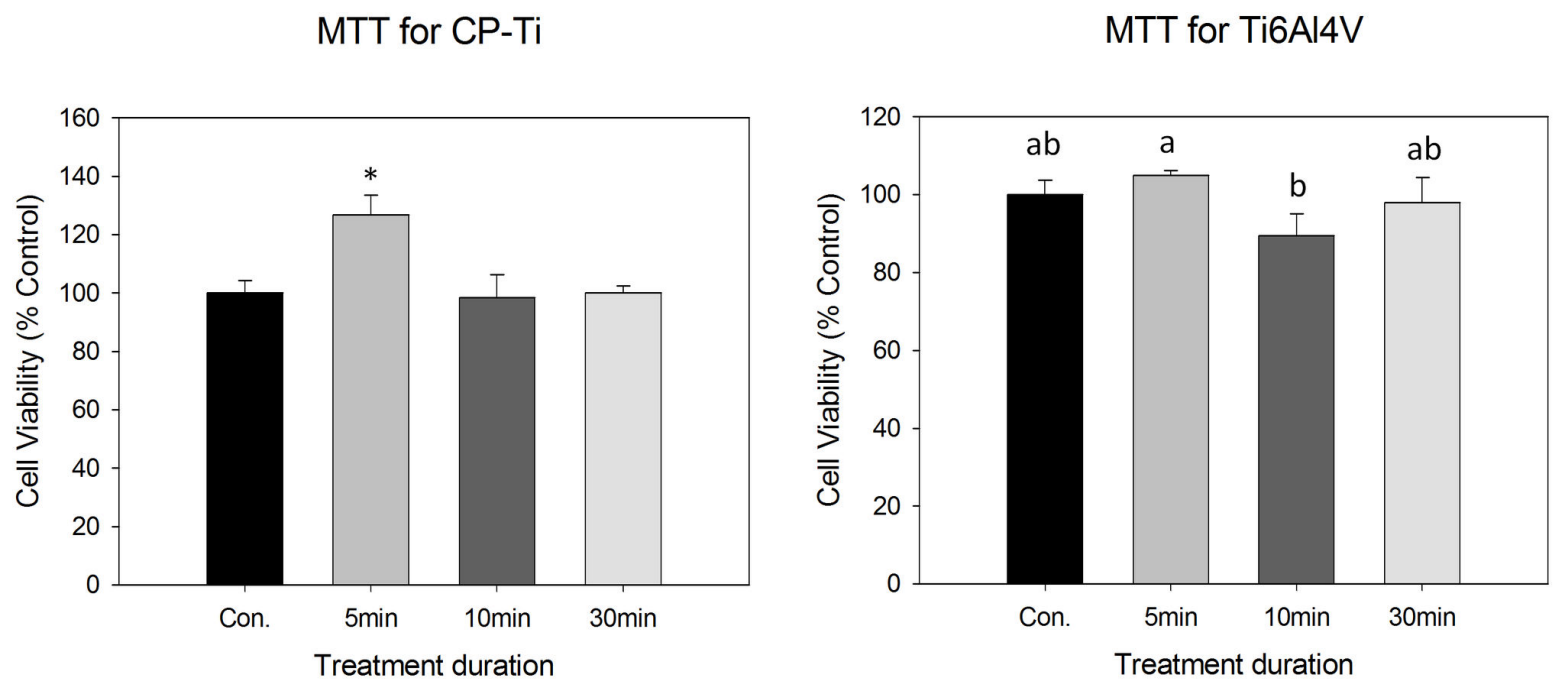
The results of MTT assay of CP-Ti and Ti6Al4V. In CP-Ti groups, star sign means significant difference; as well as Ti6Al4V groups, different letter meant statistic different. (*p* < 0.05).

The biocompatibility for CP-Ti and Ti6Al4V metal discs is shown in Figure 5. Within both groups, the metal discs in the OPT(5) group showed the best results of cell survival rate. Moreover, in the CP-Ti group, the MTT outcome of titanium discs in OPT(5) was significantly higher than other groups. 

**Figure 6:**
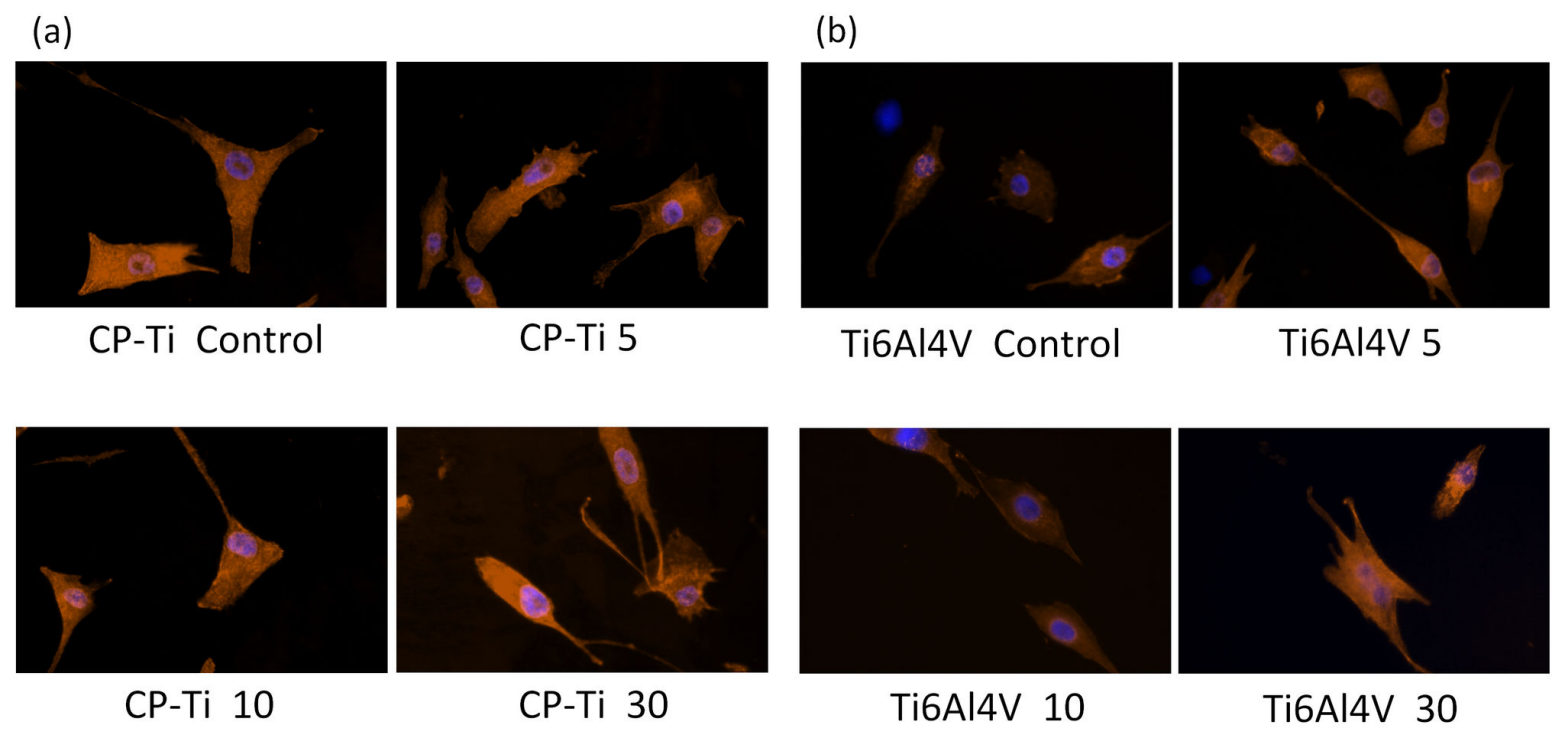
The F-actin immunofluorescence staining of MG-63 cell line cultured on CP-Ti and Ti6Al4V (200x). (a) is CP-Ti, and (b) is Ti6Al4V. The blue ovoid to round dots was the portion of cell nuclei. The cell shape of CP-Ti Control was polygonal, as well as spindle shape of other groups. All cells cultured on Ti6Al4V displayed spindle shape.

From the F-actin immunofluorescence staining of CP-Ti and Ti6Al4V, the number of osteoblasts in the OPT(5) group was greater than in other groups (Figure 6). The cytoskeleton of MG-63 in the control group of CP-Ti displayed a polygonal shape. More spindled-shape cells were observed in OPT(5), OPT(10) and OPT(30) than in the control. The cell morphology of the OP treated Ti6Al4V groups revealed a spindle shape.

## Discussion

The RMS roughness of both CP-Ti and Ti6Al4V significantly increased (*p* < 0.05) after OPT for 5 min and then decreased as the treatment time increased. In order to understand the morphological change during the OPT, we used a sputtered Ti surface of granular microstructure treated with OP and then analyzed the surface using an AFM. From the section analysis of sputtered Ti (Figure 2), the granular microstructure of sputtered Ti surface sharpened in the first 5 min and then the sharpened microstructure smoothed down after longer OPT (10 and 30 min). Although the morphology changes of both CP-Ti and Ti6Al4V due to OPT were not distinct, the trend of morphological variation is similar to that of sputtered Ti.

Hydrophilicity of materials measured by contact angle test is dependent on both the composition and the roughness of the surface. Generally, if the roughness difference between two surfaces of the same chemical composition is higher than tens of nanometers, it obviously affects the result of the contact angle test. During the process of OPT, the variation of the sample’s roughness was less than 1 nm, which should not distinctly affect the water contact angle. Therefore, the change of surface composition donated the water contact angle of titanium sample’s surface in this research. From Figure 3, the Ti 2p_3/2_ intensity of hydrophilic TiOH (457.5 ev) of 3 kinds of titanium samples obvious decreased after OPT for 5 min and thus, the surface has become hydrophobic. However, both the hydrophilicity and the XPS spectra of each substrate’s sample became stable after 5 min OPT. This phenomenon indicates that OPT for more than 5 min does not make an obvious difference on the chemical composition of the sample’s surface. Although the chemical composition of sample’s surface becomes stable with a 5 min OPT, the surface roughness starts to decrease with longer OPT. We suggest the following mechanism: Initially, in first 5 min, the bombardment of energetic OP particles performs effective oxidation of titanium to form TiO_2_ which increases the surface roughness. After the titanium oxide layer has been formed, the oxidation rate of titanium becomes stable and the effect of the OP bombardment is in smoothing the surface. Therefore, the surface roughness starts to decrease after 5 min OPT while the chemical composition is stable.

Our results demonstrate that OP treatment on titanium surfaces could improve the biocompatibility as seen in other studies. The plasma immersed titanium rods that were later implanted had a higher biochemical pull-out force and complete osseointegration was found in histological examinations [12]. In our study, both in the CP-Ti and Ti6Al4V groups, the MG-63 cell growth indicated superior biocompatibility in OPT(5) groups (Figure 5).

The surface properties, including surface roughness, free energy and tomography, play a key factor for cellular adhesion and proliferation [6,35-39]. In this study, surface modification by OP provided better condition of cell spreading and growth, especially after 5 min OPT. Duske et al. pointed out that Ar-plasma with a small portion of oxygen is favorable to cell response [21]. Even the chemical composition was the same, the cell adhesion, spread, and proliferation of preosteoblasts were enhanced by changing the micro- and nano-topographies of the titanium surface *in vitro* study [38]. The expression and mineralization of osteoblasts were also improved in rough implant surface [37]. *In vivo* studies, the osteoblasts were stimulated, and higher alkaline phosphatase (ALP) activities were noted by the rough surface in rats. Moreover, the gene expression of ALP, bone sialoprotein and osteocalcin were also increased [39]. Our study indicates that surface roughness significantly affects cell adhesion and vitality. The OPT(5) surface showed the greatest roughness and also the highest cell survival rate. However, the effect of surface roughness in nanoscale on cell growth is still controversial. According to Cai et al., the surface roughness does not influence the osteoblast proliferation and cell viability in the range of 2 to 21 nm [40]. Similar results were seen in a study by Giljean et al [41]. Ponsonnet et al. pointed out that the roughness threshold to provide better surface for cell growth might be between 80 to 100 nm [35]. The results of Sugita et al. demonstrated that modulating the surface chemistry and morphology with a super-thin TiO_2_ coating (6.3 nm in thickness) enhances cell adhesion, gene expression, and ALP activity [42].

In conclusion, oxygen plasma treatment modifies the surface of CP-Ti and Ti6Al4V, also improving biocompatibility. The surface roughness increased when the metal was treated for 5 min with OP, but then decreased in the 10 and 30 min treatments. The sharp pattern was observed on the surface of discs treated with OP for 5 min in AFM. In the XPS analysis, the intensity of Ti_metal_ (454.0 ev), TiOH (457.5 ev), TiO (454.6 ev) and Ti_2_O_3_ (456.8 ev) of the samples treated by OP decreased, which demonstrates that oxygen plasma treatment increases the intensity of TiO_2_ (459.0 ev). The water contact angle was also increased after oxygen plasma treatment. Within both groups, the discs treated for 5 min showed the best cell survival rate results. Moreover, in the Ti group, the MTT outcome of titanium discs treated for 5 minutes was significantly higher than in the other groups. The F-actin stain revealed that most of the cells displayed spindle or polygonal shapes. The technique of oxygen plasma was effective for surface modifications and resulted in favorable cell growth. 
